# Editorial: Application of novel biomarkers and natural compounds in precision oncology

**DOI:** 10.3389/fcell.2026.1783190

**Published:** 2026-02-04

**Authors:** Yuqing Li, Meng Zhang, Zhao Yang

**Affiliations:** 1 Department of Experiment and Research, South China Hospital of Shenzhen University, Shenzhen, Guangdong, China; 2 Department of Urology, The First Affiliated Hospital of Anhui Medical University, Hefei, Anhui, China; 3 College of Life Science and Technology, State Key Laboratory of Green Biomanufacturing, Innovation Center of Molecular Diagnostics, Beijing University of Chemical Technology, Beijing, China

**Keywords:** biomarkers, immunotherapy, machine learning, multi-omics, natural compounds, precision oncology

Precision oncology represents a fundamental shift in cancer management, shifting away from non-specific therapies toward strategies tailored according to the distinct molecular and genetic profiles of individual patients. This paradigm is fundamentally supported by two pillars: the identification of robust biomarkers for accurate risk stratification and the development of novel therapeutic agents-particularly those derived from natural sources—characterized by high efficacy and minimal off-target toxicity. This convergence of molecular diagnostics and targeted intervention is essential for overcoming the limitations of conventional systemic treatments, such as multidrug resistance and severe side effects associated with traditional chemotherapy. Furthermore, the integration of multi-omics data has enabled a more granular understanding of the heterogeneous tumor landscape. The Research Topic “Application of Novel Biomarkers and Natural Compounds in Precision Oncology” brings together ten articles that collectively expand current knowledge in these domains, offering compelling evidence for the potential clinical utility of emerging biomarkers and the mechanistic basis of natural-derived anti-cancer agents, thereby providing new avenues for the development of personalized treatment regimens.

Natural compounds continue to constitute a rich and indispensable reservoir for therapeutic innovation. Several studies within this Research Topic elucidate in detail the complex mechanisms by which these compounds reprogram the tumor microenvironment. For instance, Xie et al. employed a multi-omics approach to demonstrate that Curcumol exerts its anti-tumor effects in endometrial carcinoma through direct modulation of the Progesterone Receptor (PGR) and Ribosomal Protein S6 Kinase A1 (RPS6KA1), effectively suppressing cell proliferation and inducing apoptosis in malignant tissues. Their network pharmacology analysis further revealed that these interactions interfere with cell cycle progression and DNA repair mechanisms. Additionally, Cao et al. provided both experimental and computational evidence that Curcumin modulates inflammation signaling in pancreatic cancer through regulation of the IL1B pathway, specifically identifying the IL10RA and NLRP3/TLR3 axis as key regulatory nodes in this process. The study emphasizes that curcumin acts not only as a cytotoxic agent but also as a potent immunomodulator capable of reshaping the pro-tumorigenic inflammatory milieu. Beyond traditional natural extracts, Wang et al. introduced a novel synthetic derivative, the gold(I) complex TRI-03 derived from *Toona sinensis*. This compound acts as a dual-inhibitor of TrxR1 and XIAP, thereby inducing pyroptosis in melanoma cells, a finding that suggests a promising strategy to bypass the drug resistance commonly seen in conventional apoptotic pathways. The discovery of TRI-03 highlights the therapeutic potential of targeting redox homeostasis alongside anti-apoptotic proteins to trigger inflammatory cell death.

Complementing therapeutic discovery, accurate patient stratification is equally essential for clinical decision-making in precision oncology. Through metabolic subtyping, Wang et al. identified a distinct high-risk subgroup of prostate cancer patients and highlighted PDIK1L as a biomarker that drives resistance to PARP inhibitors, offering a molecular rationale for selecting patients who would benefit most from specific metabolic interventions. This work underscores how metabolic rewiring dictates pharmacological sensitivity in urological malignancies. Similarly, addressing the complexities of bladder cancer, Huang et al. constructed a risk-stratification model based on oxidative stress-related genes, providing a predictive framework for both survival outcomes and responses to immunotherapy. By utilizing advanced machine learning algorithms to refine their gene signatures, they facilitated the identification of optimal candidates for immune checkpoint blockade therapy based on the individual’s oxidative status. At a broader pan-cancer level, Dong et al. investigated the tumor-suppressive role of Chordin-Like 1 (CHRDL1), demonstrating its ability to inhibit epithelial-mesenchymal transition via the TGF-β pathway, with particular relevance to lung adenocarcinoma. Their findings suggest that CHRDL1 expression could serve as a vital indicator of metastatic potential across multiple solid tumors. Furthermore, the importance of the spatial tumor microenvironment was clearly illustrated by Wu et al., who characterized the interaction between cancer-associated fibroblasts and macrophages in breast cancer. Utilizing spatial transcriptomics, they revealed how the physical distribution and co-localization of these boundary cell features correlate directly with disease progression and therapy response, moving beyond the limitations of bulk genomic analysis.

Hepatocellular carcinoma (HCC), a malignancy characterized by pronounced heterogeneity and substantial therapeutic resistance, is the focus of two comprehensive reviews. As liquid biopsy technologies increasingly enter clinical practice for cancer screening and monitoring, Zhao et al. critically evaluated the clinical utility of circulating blood-based biomarkers, ranging from conventional serum proteins to emerging modalities such as circulating tumor DNA and exosomes. They emphasized the necessity of moving toward integrated multi-marker panels to overcome the sensitivity limitations of single-marker tests. In parallel, Chen et al. explored the gut-liver axis, proposing the gut microbiome as a novel precision biomarker that could substantially improve the prediction of responses to immune checkpoint inhibitors. Their review highlights the potential of microbiome-based interventions, such as fecal microbiota transplantation or targeted probiotics, as an adjunct to systemic therapy to enhance anti-tumor immunity. Finally, it is worth emphasizing that the computational methodologies underpinning these oncological advances exhibit broad applicability beyond the field of oncology. Zhou et al. exemplified this by applying weighted correlation network analysis (WGCNA) and machine learning algorithms to identify progression-related genes in chronic kidney disease. Their study provided a robust bioinformatic pipeline that successfully identified basement membrane-associated genes as critical prognostic factors. Although focused on a non-oncologic condition, their study underscores the universality of these advanced bioinformatics tools in unraveling complex disease mechanisms.

Collectively, the contributions to this Research Topic underscore the dynamic synergy between wet-lab validation and dry-lab computation analytics in contemporary oncology research. The integration of these multidisciplinary approaches—from the discovery of natural derivatives to the spatial mapping of the tumor microenvironment—is critical for the next-generation of clinical trials. By bridging the gap between fundamental molecular research and clinical application, these studies move us closer to a future where cancer therapy is not only precise but also highly personalized. We anticipate that these insights into novel biomarkers and natural compounds will inspire further translational research, and ultimately facilitate advancements in prognostic assessment and personalized therapeutic strategies for cancer patients worldwide.

